# Gut health and serum growth hormone levels of broiler chickens fed dietary chitin and chitosan from cricket and shrimp

**DOI:** 10.3382/ps/pey419

**Published:** 2018-09-27

**Authors:** E B Ibitoye, I H Lokman, M N M Hezmee, Y M Goh, A B Z Zuki, A A Jimoh, A Danmaigoro, N Pilau Nicholas

**Affiliations:** 1 Department of Veterinary Pre-Clinical Science Faculty of Veterinary Medicine, Universiti Putra Malaysia 43400 UPM, Serdang Selangor Darul Ehsan, Malaysia; 2 Department of Theriogenology and Animal Production, Faculty of Veterinary Medicine, Usmanu Danfodiyo University P.M.B 2346, Sokoto, Nigeria; 3 Department of Veterinary Anatomy, Faculty of Veterinary Medicine, Usmanu Danfodiyo University, P.M.B 2346, Sokoto, Nigeria; 4 Department of Veterinary Medicine, Faculty of Veterinary Medicine, Usmanu Danfodiyo University, P.M.B 2346, Sokoto, Nigeria

**Keywords:** broiler chicken, chitin, chitosan, gene expression, intestinal nutrient transporter

## Abstract

Growth hormones (GH) alone does not explain the growth rate in the chicken as growth in an animal is multi-factorial. Normal morphology of the intestinal villus and crypt, with adequate regulation of intestinal nutrient transporters, is essential to a healthy gut. Nutrition plays a significant role in gut health management, but information on the effect of dietary chitin and chitosan on gut morphology, gene expression of nutrient transporter, and serum levels of GH in broiler chickens is scanty. Thus, this study aimed at evaluating the comparative effect of dietary chitin and chitosan from cricket and shrimp on the small intestinal morphology, relative gene expression of intestinal nutrient transporters and serum level of GH in the broiler. A total of 150 day-old male Cobb500 broiler chicks were randomly allotted to one of the five treatment groups (n = 30). Treatment 1 was fed basal diet only, treatments 2 to 5 were fed a basal diet with 0.5 g cricket chitin, cricket chitosan, shrimp chitin, and shrimp chitosan, respectively, per kg diet. At days 21 and 42, duodenal and jejunal samples were assessed for structural morphology and jejunum for the relative gene expression of PepT1, EAAT3, SGLT1, and SGLT5 using quantitative real-time PCR. Results bared that dietary cricket chitosan and shrimp chitosan significantly (P < 0.05) improved jejunal villus height and reduced crypt depth without improving the body weight (BW). The gut morphology of birds under cricket chitin was poor and significantly (P < 0.05) different from other treated groups. Both the dietary chitin and chitosan at day 21 and only dietary chitosan at day 42 significantly (P < 0.05) down-regulated the relative mRNA expression of PepT1, EAAT3, SGLT1, and SGLT5 of broiler chickens. Treated groups differ non-significantly at both phases, while cricket chitin numerically increased the relative expression of PepT1, EAAT3, and SGLT1. Therefore, the potential of cricket chitin to improve BW and to up-regulate nutrient transporters is worthy of further exploration.

## INTRODUCTION

The gut health is important in the maintenance of efficient production, with the primary role of digestion and absorption of nutrient (Sobolewska et al., 2017). Nutrient absorption is critical to animal growth and productivity which partly rely on the ability of the gut to digest and absorb ingested macromolecules (Sangild, 2003). More so, the eventual manifestation of growth is multi-factorial, and that growth hormones (**GH**) alone does not explain the growth rate in the chicken (Beccavin et al., 2001). However, among the hormones required to support normal growth in chickens are growth hormones (**GH**) and insulin-like growth factor-1 (**IGF-1**) (Scanes, 2009). In addition, growth in animals has been closely related to the mRNA expression levels, this is because intestinal nutrient transporters are needed for the uptake of products of digestion into the circulation (McCracken and Edinger, 2013). Glucose is transported from the lumen of the small intestine across the brush border membrane into the enterocyte mainly by the sodium-dependent glucose transporter (**SGLT1**), while the facilitative glucose transporter (GLUT5) facilitates the passive transport of fructose into enterocytes. Proteins in the small intestine are enzymatically broken down into peptides and free amino acids and are transported either as free amino acids or as small peptides by a variety of amino acid transporters or the peptide transporter, (PepT1), respectively. Free glutamate and aspartate are transported across the brush border membrane of the enterocytes by the excitatory amino acid transporter (**EAAT3**) (Mott et al., 2008). According to McCracken and Edinger (2013), one of the regulatory pathways of gene expression of the intestinal nutrient transporter is the response to the availability of nutrient; thus nutrient reduction elicits the adaptive up-regulation of transporters for the little nutrients. Studies have revealed that acute undernutrition up-regulates the mRNA expression of PepT1 (Sampaio et al., 2016). Furthermore, a greater amount of PepT1 and EAAT3 mRNA has been associated with the poor dietary protein quality (Osmanyan et al., 2018).

Studies have been concentrated on the microbial aspect of the poultry gut health (Jimoh et al., 2017), whereas, normal gut morphology and integrity are significant in the maintenance of intestinal microbial homeostasis in the prevention of infection and promoting digestion and absorption of nutrients (Gomes et al., 2014). Earlier histological studies of the intestines had revealed that dietary components could affect the intestinal morphology in chickens (Incharoen et al., 2010). Recently, Elsamanoudy et al. (2016), reported that the interaction between nutrition, metabolism, and gene expression is required for the preservation of body homeostasis. Also, according to Meibom et al. (2005) chitin or its derivatives was reported to influence the expression of 41 genes of *Vibrio cholera.* Dietary chitin (CT), a *β-(1–4)-N-*acetyl-d-glucosamine, and chitosan (**CS**), a *β-(1–4)-*linked d-glucosamine isolated from crustacean, are known to affect growth performance of broiler chickens (Kobayashi et al., 1996; Nuengjamnong and Angkanaporn, 2018), however, their mechanism of action is still a topic of concern. There is a gross dearth of information on the effect of dietary CT and CS on the gut morphology, mRNA expression of intestinal nutrient transporters and growth hormone (GH) in broiler chickens. Moreover, comparative effect of dietary cricket and shrimp CT and CS on broiler gut health has not been studied. This study was therefore conducted to compare the effect of cricket and shrimp CT and CS on gut morphology, relative gene expression of nutrient transporters, and serum levels of GHs in broiler chickens.

## MATERIALS AND METHODS

### Experimental Birds and Feeding

This study obtained ethical clearance (UPM/IACUC/AUP-R025/2017) from the Institutional Animal Care and Use Committee of the Universiti Putra Malaysia. Experimental broiler chickens were managed and sampled according to approved guidelines (Federation Animal Science Societies, 1999). This study was carried out at the Broiler Chicken Unit, Poultry House of the Department of Animal Science, Faculty of Agriculture, Universiti Putra Malaysia. Using a completely random design, 150 day-old male Cobb500 broiler chicks, with an average weight of 48.93 g, were allotted one of the five dietary treatment groups (n = 30) in triplicate of 10 birds/replicate. Antibiotic-free basal diets were obtained from poultry feed supplier, processed into experimental diets, and were fed *ad libitum* in the mash form to the experimental broiler chickens for 42 days. Birds in Treatment 1 (T1) were given basal diet alone (Control), while birds in treatments 2, 3, 4, and 5 (T2, T3, T4 and T5), were served a basal diet with 0.5 g/kg of cricket chitin (**CCT**), cricket chitosan (**CCS**) (Ibitoye et al., 2018b), shrimp chitin (**SCT**) and shrimp chitosan (**SCS**) (Sigma-Aldrich USA) (Tables 1 and 2). Birds were raised on wire-floored cages, placed in an open-sided pen, and the study was divided into starter (days 0 to 21) and grower (days 22 to 42) phases. Vaccination and brooding were done according to Al-Aqil and Zulkifli (2009).

**Table 1.** The composition of broiler starter diet containing cricket and shrimp and CS.

	Treatments				
Ingredients (kg)	T1	T2	T3	T4	T5
Corn	50.32	50.32	50.32	50.32	50.32
Soybean meal	39.02	39.02	39.02	39.02	39.02
Palm oil	6.67	6.67	6.67	6.67	6.67
Dicalcium phosphate	1.62	1.62	1.62	1.62	1.62
Limestone	1.22	1.22	1.22	1.22	1.22
Sodium chloride	0.50	0.50	0.50	0.50	0.50
Vitamin premix	0.05	0.05	0.05	0.05	0.05
Mineral premix	0.10	0.10	0.10	0.10	0.10
L-Lysine, HCL	0.15	0.15	0.15	0.15	0.15
DL methionine	0.20	0.20	0.20	0.20	0.20
Calculated nutrient composition					
ME (kcal/kg)	3130	3130	3130	3130	3130
Crude protein (%)	22.2	22.2	22.2	22.2	22.2
Available phosphorus (%)	0.45	0.45	0.45	0.45	0.45
Methionine + cysteine (%)	0.90	0.90	0.90	0.90	0.90
Lysine (%)	1.22	1.22	1.22	1.22	1.22
Test materials (g/kg)					
CCT	–	0.50	–	–	–
CCS	–	–	0.50	–	–
SCT	–	–	–	0.50	–
SCS	–	–	–	–	0.50

**Table 2.** Composition and calculated nutrient contents of broiler grower diet containing cricket and shrimp and CS.

	Treatments				
Ingredients (kg)	T1	T2	T3	T4	T5
Corn	58.70	58.70	58.70	58.70	58.70
Soybean meal	31.68	31.68	31.68	31.68	31.68
Palm oil	6.15	6.15	6.15	6.15	6.15
Dicalcium phosphate	1.18	1.18	1.18	1.18	1.18
Limestone	1.31	1.31	1.31	1.31	1.31
Sodium chloride	0.42	0.42	0.42	0.42	0.42
Vitamin premix	0.05	0.05	0.05	0.05	0.05
Mineral premix	0.10	0.10	0.10	0.10	0.10
L-Lysine, HCL	0.11	0.11	0.11	0.11	0.11
DL methionine	0.15	0.15	0.15	0.15	0.15
Calculated nutrient composition					
ME (kcal/kg)	3200	3200	3200	3200	3200
Crude protein (%)	19.5	19.5	19.5	19.5	19.5
Available phosphorus (%)	0.35	0.35	0.35	0.35	0.35
Methionine + cysteine (%)	0.79	0.79	0.79	0.79	0.79
Lysine (%)	1.03	1.03	1.03	1.03	1.03
Test materials (g/kg)					
CCT	–	0.50	–	–	–
CCS	–	–	0.50	–	–
SCT	–	–	–	0.50	–
SCS	–	–	–	–	0.50

### Gut Morphology

At the end of starter and grower phases, feeds were withdrawn from birds overnight, then two birds were randomly selected per replicate, weighed, and sacrificed using Malaysian Standard (MS) 1500:2009. Two-centimeter segments from the middle part of the duodenum and jejunum were removed, flushed with physiological saline, and fixed in 10% buffered formalin until further processing. Tissue processing and staining were done according to the method of Zakariah et al. (2016). The tissues were examined under different magnifications of the light microscope for the histological view to measure villus height (**VH**) and crypt depth (**CD**) and were photographed using a Moticam 2.0 MP camera. The distance from the tip of the villus to the villus-crypt junction represents VH, while CD was defined as the depth of the invagination between adjacent villi (Tenesa et al., 2016).

### Gene Expression Analysis

Gene expression analyses were carried out from jejunal samples at days 21 and 42 as earlier described by Ebrahimi et al. (2015). A 2 cm tissue of mid jejunum was excised and submerged in RNAlater solution (Invitrogen) and was stored at -20°C before further analysis. Total RNA was isolated using Total RNA Mini Kit (Tissue) (Geneaid Biotech Ltd.) following kit's manufacturer instructions. A spectrophotometer (infinite M200PRO TECAN) at a wavelength of 450 nm ± 2 nm was used to determine RNA purity and concentration, then the RNA was stored at -80°C until further use. Reverse transcription was performed using ReverTra Ace qPCR RT Master Mix with gDNA remover kit (TOYOBO Bio-Technology, CO., LTD), followed by a quantitative real-time PCR using a PCR machine (Bio-Rad CFX96). Two and a half microliters of cDNA was added into a 0.2 ml PCR tube, then 10 μl KOD SYBR qPCR Mix 17.5 μl of a real-time PCR master mix [Per reaction: 10 μl KOD SYBR qPCR Mix (Toyobo); 1 μl each of a 0.2 μM forward primer and reverse primer; 5.5 μ distilled water] was added to each PCR tubes. PCR cycling conditions included pre-denaturation at 98°C for 2 min followed by 45 cycles of denaturation at 98°C for 10 secs, annealing at 60°C for 10 secs, and extension at 68°C for 30 secs. Genes analyzed were oligopeptide transporter (PepT1), the excitatory amino acid transporter (**EAAT3**), Na^+^-dependent glucose and galactose transporter (SGLT1) and Na^+^-independent fructose transporter (SGLT5). The endogenous control used were glyceraldehyde-3-phosphate dehydrogenase (**GAPDH**) and Beta-actin (β-actin). Gene-specific primers designed and reported by Faseleh Jahromi et al. (2017) were used in this study (Table 3). All plates wells were analyzed independently with a software (Bio-Rad CFX Manager 3.1) provided with the Bio-Rad CFX96 Real-Time PCR machine using the Auto function. To determine relative gene expression, ΔΔCT method was employed (Livak and Schmittgen, 2001), where ΔCT equals CT value of the target gene minus that of the reference genes (GAPDH and β-actin), subsequently finding the average. The ΔΔCT is the difference between the ΔCT of treated samples and that of the control. Data are presented as fold change expression in the target gene of a treated sample compared to the control samples.

**Table 3.** Primer sequences (5΄ → 3΄) used in real-time PCR.

Name	Forward primer	Reverse primer	Product size (bp)	Annealing temp (°C)
	Intestinal nutrient transporters			
SGLT1	TGTCTCTCTGGCAAGAACATGTC	GGGCAAGAGCTTCAGGTATCC	229	60.2
SGLT5	ATACCCAAGGTAATAGTCCCAAAC	TGGGTCCCTGAACAAATGAAA	75	60
PepT1	CCCCTGAGGAGGATCACTGTT	CAAAAGAGCAGCAGCAACGA	205	60
EAAT3	TGCTGCTTTGGATTCCAGTGT	AGCAATGACTGTAGTGCAGAAGTAATATATG	79	60
GAPDH	GCCGTCCTCTCTGGCAAAG	TGTAAACCATGTAGTTCAGATCGATGA	128	60
β actin	CAACACAGTGCTGTCTGGTGG	ATCGTACTCCTGCTTGCTGAT	205	55

### Serum Growth Hormone Assay

Immediately after sacrifice of the birds, blood samples were collected in labelled plain tubes, and serum was harvested for the analysis of chicken growth hormone (**CGH**) using chicken growth hormone (**CGH**) ELISA Kit (Elabscience), while chicken insulin-like growth factor 1 (IGF-1) was evaluated using chicken insulin-like growth factor 1 (IGF-1) ELISA kit (Elabscience). The optical density (**OD**) of GH, were measured using a spectrophotometer (infinite M200PRO TECAN) at a wavelength of 450 nm ± 2 nm. The OD value is proportional to the concentration of GHs. Then the concentration of GHs in the samples was calculated by comparing the OD of the samples to the standard curve.

### Statistical Analysis

Results were presented in charts and as average values ± SEM. All data were analyzed using one-way ANOVA to test the foremost effect of the treatment, while the least significant difference (LSD) analysis was used to relate differences between means. In addition, student t-test was used to analyze them for the significant difference in the results of gene expression study using SPSS (IBM, 2013). The values with (P < 0.05) were said to be significant.

## RESULTS

### Gut Morphology of Experimental Chickens

This study compared the effect of dietary CT and CS from cricket and shrimp at 0.5 g/kg feed on the jejunal and duodenal height and depth of villus and crypt respectively. It was revealed that dietary CT and CS significantly affected the CD of broiler (P = 0.009 and 0.003) at the starter and grower phases, respectively (Table 4). At age 21 days, dietary CCS and SCT significantly (P < 0.05) reduce CD, with the deepest CD observed in the control group. In the same vein, a similar trend was observed at day 42; however, birds in T2 (CCT) group had the deepest CD. The VH: CD ratio of the broiler chickens under this investigation was presented in Table 4. It was observed that our dietary treatments significantly affected the VH: CD ratio at both ages 21 (P = 0.015) and 42 (P = 0.011) of broiler chickens. At both day 21 and 42, VH: CD ratio significantly (P < 0.05) increased in birds fed the diet with SCT more than those of control and CCT groups that have the lowest ratios (Table 4). Also, dietary CCS and SCS groups quadratically (P > 0.05) improved VH: CD ratio more than the control birds and significantly (P < 0.05) more than the CCT group. At the end of the study, CD and VH: CD ratios of birds fed dietary CCT significantly (P < 0.05) different from other groups except for the control.

**Table 4.** Gut morphology of broiler chickens fed 0.5 g CCT, CCS, SCT, and SCS/kg diet (Mean ± SEM).

	BW d 21	VH d 21	CD d 21	VH:CD d 21	BW d 42	VH d 42	CD d 42	VH:CD d 42
Jejunum								
T1	734.66 ± 14.25	69.43 ± 0.58	28.30 ± 2.94^a^	2.50 ± 0.22^b,c^	2670.14 ± 60.89^a^	79.63 ± 5.62	20.93 ± 2.70^a,b^	3.90 ± 0.43^b,c^
T2	733.00 ± 7.81	65.19 ± 0.35	28.10 ± 0.36^a^	2.32 ± 0.02^c^	2565.42 ± 47.02^a,b^	68.22 ± 1.76	25.48 ± 1.86^a^	2.71 ± 0.24^c^
T3	724.33 ± 6.39	52.73 ± 2.69	10.73 ± 0.25^b^	4.97 ± 0.37^a,b^	2359.35 ± 16.60^c^	71.92 ± 7.09	15.36 ± 2.05^c^	4.91 ± 0.28^a,b^
T4	748.67 ± 9.39	61.76 ± 6.97	11.07 ± 0.72^b^	5.56 ± 0.45^a^	2518.99 ± 15.60^b^	81.55 ± 8.32	14.64 ± 0.68^c^	5.58 ± 0.46^a^
T5	749.00 ± 16.09	70.46 ± 3.94	15.11 ± 1.13^a,b^	4.79 ± 0.29^a,b^	2514.35 ± 21.77^b^	68.4 ± 3.46	15.77 ± 1.55^b,c^	4.64 ± 0.55^a,b^
P-value	0.511	0.064	0.009	0.015	0.002	0.466	0.003	0.011
Duodenum								
T1	734.66 ± 14.25	66.03 ± 6.164	15.90 ± 1.869	4.41 ± 0.571	2670.14 ± 60.89^a^	79.39 ± 8.819	18.37 ± 2.625	4.68 ± 0.412
T2	733.00 ± 7.81	53.38 ± 1.854	11.61 ± 0.84	4.82 ± 0.368	2565.42 ± 47.02^a,b^	61.30 ± 8.120	11.94 ± 0.866	5.24 ± 0.414
T3	724.33 ± 6.39	60.66 ± 9.03	12.91 ± 2.121	4.80 ± 0.291	2359.35 ± 16.60^c^	59.14 ± 7.991	14.94 ± 3.131	4.11 ± 0.361
T4	748.67 ± 9.39	70.08 ± 1.273	10.75 ± 0.959	6.80 ± 0.915	2518.99 ± 15.60^b^	77.01 ± 9.174	19.87 ± 3.013	4.07 ± 0.351
T5	749.00 ± 16.09	58.44 ± 9.514	14.17 ± 2.120	4.27 ± 0.459	2514.35 ± 21.77^b^	58.53 ± 0.291	9.76 ± 1.312	6.33 ± 0.828
P-value	0.511	0.286	0.358	0.87	0.002	0.567	0.124	0.053

Column with different superscripts a, b, c, a, b, and b, c are significantly different (P < 0.05).

BW: body weight, VH: villus height, CD: crypt depth and VH: CD: villus height: crypt depth ratio.

### Intestinal Nutrient Transporter of Broiler Chickens

**Figure 1.**
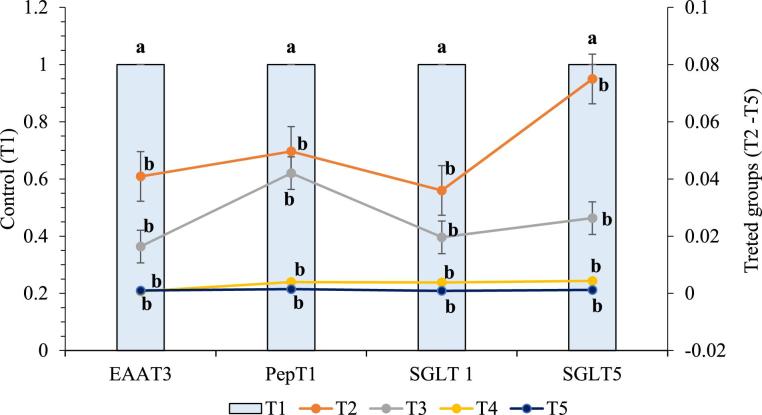
Relative mRNA expression of nutrient transporters in experimental broilers at day 21. Data with different letters a, b are significantly different (P < 0.05).

**Figure 2.**
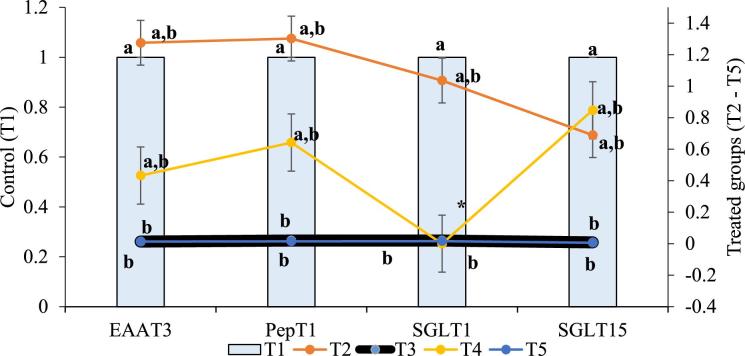
Relative mRNA expression of nutrient transporters in experimental broilers at day 42. Data with different letters a, b, a,b are significantly different (P < 0.05), while * = SGLT1 data for T4 is not available

From this study, it was observed that at both starter and grower stages of production CT and/or CS from cricket or shrimp at 0.5 g/kg diet as feed additives significantly (P < 0.05) affected PepT1, EAAT3, SGLT1, and SGLT5 of the experiment broiler chickens. At day 21, the relative gene expression of PepT1, EAAT3, SGLT1 plus SGLT5 were meaningfully (P < 0.05) down-regulated in birds fed dietary CT and/or CS i.e., T2, T3, T4, and T5 (Figure 1). At day 42, as shown in Figure 2, dietary CS significantly (P < 0.05) down-regulated the relative gene expression of PepT1, EAAT3, SGLT1, and SGLT5, while they were non-significantly affected by dietary CT (P > 0.05). Furthermore, the relative mRNA expression of PepT1, EAAT3, SGLT1, and SGLT5 differ non-significantly among the treated groups (T2–T5). However, CCT at 0.5 g/kg diet quadratically up-regulated the relative expression of PepT1, EAAT3, and SGLT1.

### Serum Growth Hormones

Table 5 presents the result of growth hormones evaluation in broiler chickens. Addition of CT and/or CS from either cricket or shrimp at 0.5 g/kg diet non-significantly affected (P > 0.05) the CGH of broilers all through the experimental period. On the other hand, the effect of dietary CT and CS/kg diet have a significant effect on the IGF-1 at day 42 (P < 0.05). When compared with the control birds, at grower phase of broiler production, dietary SCS significantly increased serum IGF-1 (21.48 ± 2.641 ng/mL) above all other treatments.

**Table 5.** Growth hormone profile of experimental birds (mean ± SEM).

	Treatments				
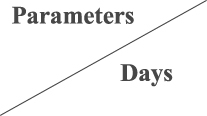	T1	T2	T3	T4	T5
CGH (ng/mL)					
day 21	5.69 ± 1.683	1.76 ± 1.719	23.30 ± 8.866	20.05 ± 9.853	16.02 ± 8.105
day 42	7.23 ± 3.395	3.66 ± 1.187	9.45 ± 2.234	8.17 ± 3.755	8.954 ± 2.170
day 1 to 42	6.46 ± 1.729	2.71 ± 1.027	16.38 ± 5.130	14.11 ± 9.418	12.49 ± 4.071
IGF-1 (ng/mL)					
day 21	8.03 ± 0.237	8.23 ± 0.437	7.98 ± 0.130	10.72 ± 1.036	8.36 ± 0.416
day 42	8.74 ± 0.185^b^	9.08 ± 0.079^b^	11.10 ± 2.485^b^	9.64 ± 0.901^b^	21.48 ± 2.641^a^
day 1 to 42	8.39 ± 0.209	8.65 ± 0.274	9.54 ± 1.313	10.18 ± 0.660	12.72 ± 4.370

Rows with different superscripts a, b are significantly different (P < 0.05).

## DISCUSSION

In this study, dietary CT and/or CS had no significant (P > 0.05) effect on the jejunal and duodenal VH of experimental chickens. This suggests that dietary CT and CS did not trigger programmed cell death, and this agreed with the findings of Nuengjamnong and Angkanaporn (2018) from a study in broiler chickens. This agreement may be due to the similarity in the physicochemical properties of CS used in both studies. Dietary CCS and SCT significantly decreased (P < 0.05) the jejunal CD of birds, while SCT significantly improved the VH: CD ratio. This contradicted the findings of Xu et al. (2013), and this might be caused by the difference in animal species and the quality of the materials used. In addition, it has been reported that pathogenic organisms which affect the physiology of the GIT could be inhibited by dietary CS, hence, improve gut morphology (Xu et al., 2012). It is therefore implied that dietary inclusion of CCS and SCT might significantly increase the absorptive capacity and improve the gut health (Montagne et al., 2003), and consequently promote growth (Marković et al., 2009). Contrary to this speculation, but in accordance with de Verdal et al. (2010), by the end of this experiment, the inclusion of CCS, SCT, and SCS, although decreased CD and increased VH: CD ratios, did not improve the BW of experimental broilers in this study. This maybe because dietary CT and CS are known hypocholesterolemic agents (Ibitoye et al., 2018a), or that dietary CCS, SCT, and SCS may have compromised feed digestion and/or nutrient absorption. Our earlier work on cricket and shrimp CT and CS revealed that CCT is of better quality and of purer form than SCT (Ibitoye et al., 2018b). However, this study could not explain why SCT had a significantly better effect on the jejunal CD and VH: CD ratio of broilers than CCT.

It has been established that the availability of nutrient is one of the pathways that regulate gene expression (McCracken and Edinger, 2013). In the periods of nutrient unavailability, there is up-regulation of intestinal nutrient transporters for the scarce nutrients (Osmanyan et al., 2018). This study reported a significant down-regulation of EAAT3, PepT1, SGLT1, and SGLT5 by dietary CT and CS at day 21. This indicated that CT and CS from either cricket or shrimp might have no significant adverse effect on feed intake, feed quality, digestion, and nutrient availability in the body system. However, the down-regulation of nutrient transporters by dietary CT and CS in this study might be because CT and CS negatively interfere with the substrates of these transporters (Adibi, 2003). Furthermore, epigenesis, for instance, histone acetylation, cytosine methylation of DNA, micro RNAs (miRNAs), and noncoding RNAs have been linked to the alteration in the gene expression process rather than an alteration in the DNA sequence (Elsamanoudy et al., 2016). Documented outcomes and proofs, proposes that methylation of DNA, modification of histone, and miRNAs could have integrated effect in the regulation process of gene expression (Kim et al., 2008). For instance, histone acetylation must occur before transcription can be initiated (Tümer et al., 2013), whereas histone acetylation is known to increase mRNA expression (Gallinari et al., 2007). Chitin is a *β-N-*acetylglucosamine, which under the influence of histone acetyltransferase could cause the acetylation of histone, consequently resulting in the non-significant effect of CT on relative mRNA expression of PepT1, EAAT3, SGLT1, and SGLT5 in broiler chickens. Chitosan, on the other hand, is a *β-D-*glucosamine, with little or no acetyl groups; this may have limited the level of histone acetylation and hence may have resulted in the significant down-regulation of mRNA intestinal nutrient transporters in broiler chickens.

This study evaluated the effect of dietary CT and CS from shrimp and/or cricket on the serum levels of GH and IGF-1 in broiler chickens, and it was observed non-significant, except for IGF-1 at day 42. In this study, dietary CCS, SCT, and SCS at 0.5 g quadratically elevated serum GH and IGF-1 of chickens without a corresponding increase in BW. This contradicts the report of Xiao et al. (2017) that heavier BW is consistent with the higher serum concentration of IGF-1 in broiler chickens. Xiao et al. (2017) used only the basal diet, while CT and CS were factors in this study. In agreement with the present result, Buyse and Decuypere (1999) concluded that exogenous GH only slightly, if any, improve growth in the avian species. Their result was attributed to age-related changes in tissue GH-binding activity and GH-receptor mRNA expression. Therefore, one reason the high level of GH and IGF-1 in this study did not result in increased BW; might be that dietary CT and CS down-regulate the expression of GH- and IGF-1-binding mRNA receptors and activity. The higher level of GH observed in the male broiler chickens used in this study is consistent with the findings of Pampori and Bernard (1994) that plasma concentrations of GH is higher in male chickens because estrogen depresses GH synthesis (Fanciulli et al., 2009). It has been shown that dietary CT significantly elevated plasma concentration of thyroxine of chicks (Kobayashi et al., 1996). Since thyroid hormones “run the body's metabolism,” it is therefore understandable that increase in the level of these hormones will increase the body metabolism and cause depression of weight gain (Spear and Moon, 1986).

## CONCLUSION

Based on the results of the present study, although dietary CCS, SCT, and SCS significantly improved the jejunal morphology and integrity and quadratically the GH and IGF-1, they except CCT, did not improve the BW of broilers. At day 21, dietary CT and CS significantly down-regulated the mRNA expression of PepT1, EAAT3, SGLT1, and SGLT5, while at day 42, CCT up-regulated the gene expression of PepT1, EAAT3, and SGLT1 in broiler chickens. Therefore, gene expression of intestinal nutrient transporters seems to affect BW in broiler chickens more than the improved gut morphology and serum growth hormone levels. This study found no significant difference between CT and CS groups in terms of gene expression of nutrient transporters, while dietary CCT negatively affected the jejunal morphology of birds and significantly differ from other treatments. Thus, the potentials of CCT in improving BW and up-regulating gene expression of nutrient transporters of chicken may reasonably translate the potential of this compound as the nutritional tools for growth promotion in poultry. Further studies with graded doses of chitin and chitosan, with their effect on the regulation of substrates of nutrient transporters as wells as the mRNA genes expression of GH and IGF-1 receptors, are recommended.
